# Cardiotoxicity of BRAF/MEK Inhibitors: A Longitudinal Study Incorporating Contemporary Definitions and Risk Scores

**DOI:** 10.1016/j.jaccao.2023.04.004

**Published:** 2023-06-06

**Authors:** Claire Glen, Sarah Adam, Kirsty McDowell, Ashita Waterston, Yun Yi Tan, Mark C. Petrie, Caroline J. Coats, Ninian N. Lang

**Affiliations:** a School of Cardiovascular and Metabolic Health, University of Glasgow, Glasgow, United Kingdom; b Queen Elizabeth University Hospital, National Health Service Greater Glasgow and Clyde, Glasgow, United Kingdom; c Beatson West of Scotland Cancer Centre, National Health Service Greater Glasgow and Clyde, Glasgow, United Kingdom

**Keywords:** BRAF inhibitor, cancer therapy–related cardiac dysfunction, cardiotoxicity, global longitudinal strain, left ventricular ejection fraction, MEK inhibitor

## Abstract

**Background:**

Rapidly accelerated fibrosarcoma B-type (BRAF) and mitogen-activated extracellular signal-regulated kinase (MEK) inhibitors have revolutionized treatment for patients with BRAF-mutated melanoma. Although left ventricular systolic dysfunction associated with these therapies has been reported in clinical trials, the real-world incidence is poorly defined, as are risk factors for its development.

**Objectives:**

This study sought to characterize the incidence, time course, and risk factors for cancer therapy–related cardiac dysfunction (CTRCD) in patients with melanoma receiving BRAF and MEK inhibitors.

**Methods:**

Patients with melanoma treated with BRAF and MEK inhibitors at a cancer hospital network between June 1, 2017, and June 30, 2020, were included retrospectively. CTRCD was defined as mild, moderate, or severe according to International Cardio-Oncology Society (ICOS) definitions. Baseline cardiotoxicity risk stratification was performed using the Heart Failure Association/ICOS tool.

**Results:**

Of the 63 patients included, 27% developed CTRCD (17% mild and 10% moderate). No patients developed severe CTRCD or symptomatic heart failure. CTRCD occurred most frequently in patients considered to be at “low” and “medium” baseline risk of cardiotoxicity (82%). The baseline left ventricular ejection fraction and global longitudinal strain were not different in patients who developed moderate CTRCD vs those who did not. Left ventricular internal diameters in diastole and systole were larger in patients who developed moderate CTRCD compared with those who did not (left ventricular internal diameter in diastole: 4.9 ± 0.6 cm vs 4.3 ± 0.6 cm; *P =* 0.023; left ventricular internal diameter in systole: 3.3 ± 0.4 cm vs 2.8 ± 0.5 cm; *P =* 0.039).

**Conclusions:**

BRAF and MEK inhibitor–associated CTRCD is common. The utility of the Heart Failure Association/ICOS risk stratification tool appears limited in this group, and better risk prediction tools are needed. The long-term consequences of CTRCD, particularly mild CTRCD, warrant evaluation in larger prospective studies.

The introduction of targeted therapies against rapidly accelerated fibrosarcoma B-type (BRAF) and mitogen-activated extracellular signal-regulated kinase (MEK) inhibitors has revolutionized treatment for patients whose melanoma harbors a BRAF V600 gene mutation, especially when these drugs are used in combination.^1^ BRAF inhibitors include dabrafenib, vemurafenib, and encorafenib, whereas MEK inhibitors include trametinib, binimetinib, and cobimetinib. Unfortunately, these therapies are associated with cardiovascular adverse effects including left ventricular systolic dysfunction (LVSD).^2^, ^3^, ^4^ The incidence of LVSD reported in clinical trials is 2% to 12%,^3^, ^4^, ^5^ but the real-world incidence is poorly described and may be higher than this.

A recent International Cardio-Oncology Society (ICOS) consensus statement^6^ defines the spectrum of asymptomatic cancer therapy–related cardiac dysfunction (CTRCD), and these diagnostic criteria (Central Illustration) have also been adopted by the inaugural European Society of Cardiology (ESC) cardio-oncology guidelines. Mild asymptomatic CTRCD is considered to have occurred when the left ventricular ejection fraction (LVEF) remains preserved (≥50%), but the global longitudinal strain (GLS) has declined by more than 15% relative to the baseline measurement.^6^ Most prior definitions of cardiotoxicity have required a decline in LVEF to meet the criteria for CTRCD. Evidence derived from patients treated with other anticancer therapies suggests that GLS can be used to detect early subclinical left ventricular dysfunction^7^ and, through early identification, may provide an opportunity to prevent subsequent myocardial dysfunction and a significant decline in LVEF. With the addition of this more sensitive GLS-based definition, there is potential for a much larger proportion of patients to be considered as having developed cardiotoxicity. The incidence and clinical relevance of mild cardiotoxicity in patients treated with BRAF and MEK inhibitors are unclear. In particular, the incidence of a subsequent decline in LVEF or the development of symptomatic heart failure has not been defined in this group. In those patients identified to have had a decline in LVEF, the consequences of this are also not well-defined.

**Central Illustration:**
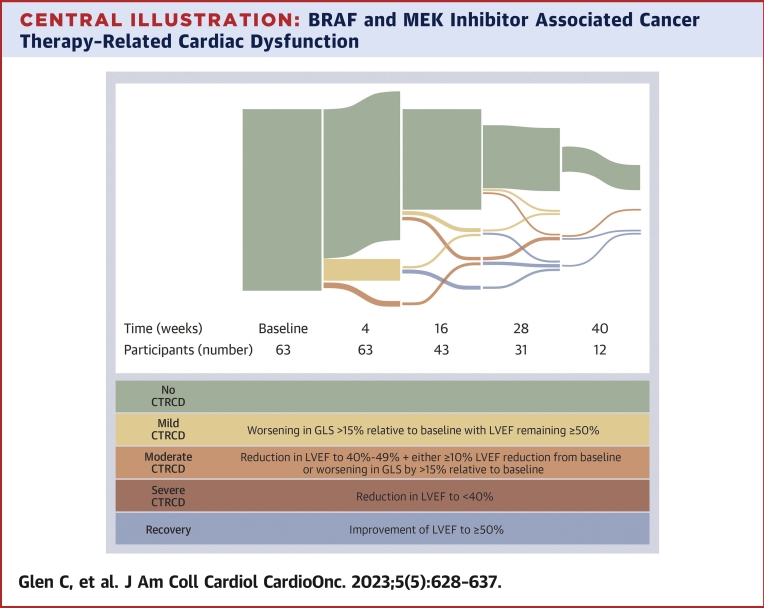
BRAF and MEK Inhibitor Associated Cancer Therapy–Related Cardiac Dysfunction A Sankey diagram detailing the proportion of participants classified as no, mild, or moderate cancer therapy–related cardiac dysfunction (CTRCD) and recovered at specified time points during treatment with rapidly accelerated fibrosarcoma B-type (BRAF) and mitogen-activated extracellular signal-regulated kinase (MEK) inhibitors, including the definitions of CTRCD. GLS = global longitudinal strain; LVEF = left ventricular ejection fraction.

Summary of Product Characteristics package inserts recommend echocardiography at baseline, after 4 weeks, and at 12 weekly intervals thereafter during treatment with BRAF and MEK inhibitors.^8^, ^9^, ^10^, ^11^, ^12^, ^13^ The ESC cardio-oncology guidelines^14^ recommend a different approach based on baseline risk. The recommended Heart Failure Association (HFA)/ICOS cardiotoxicity risk tool^15^ incorporates a wide variety of clinical parameters but has not had robust validation.

Using contemporary ICOS CTRCD definitions, we characterized the incidence, time course, and evolution of CTRCD in a cohort of patients with melanoma treated with BRAF and MEK inhibitors in a real-world setting. We aimed to improve understanding of the clinical relevance of changes in both GLS and LVEF in this patient group. We also categorized patients using the HFA/ICOS baseline cardiotoxicity risk assessment tool to assess its utility in the stratification of patients potentially at risk of BRAF and MEK inhibitor–associated cardiotoxicity.

## Methods

### Study population

We conducted a retrospective case series review of patients treated at a regional cancer hospital network (West of Scotland Cancer Network, National Health Service). Patients with melanoma treated at 12 hospitals with a BRAF inhibitor in combination with an MEK inhibitor between June 1, 2017, and June 30, 2020, were included. In this hospital network, all patients treated with BRAF/MEK inhibitors undergo echocardiography at baseline, at 4 weeks after starting treatment, and every 3 months thereafter until BRAF/MEK inhibitor treatment discontinuation. Patients without a baseline echocardiogram or a follow-up echocardiogram were excluded. This study was approved by the Caldicott Guardian for National Health Service Greater Glasgow and Clyde and the UK National Research Ethics Service (Reference 22/WM/0191).

### Data collection

All clinical data were collected from electronic patient records. Baseline data included age, sex, indication for treatment, treatment regimen, prior anthracycline exposure, prior melanoma therapy, body mass index, smoking status, history of cardiovascular disease (prior myocardial infarction, coronary artery bypass grafting, or stable angina) or cardiovascular risk factors (hypertension, diabetes mellitus, or chronic kidney disease), history of heart failure, clinic measurement of systolic and diastolic blood pressure, and cardiovascular medications. The HFA/ICOS baseline risk assessment tool was used to categorize patients considered to be at “very high,” “high,” “medium,” and “low risk” of cardiotoxicity (Supplemental Table 1).^15^ Cardiovascular adverse events were assessed retrospectively from the comprehensive electronic health record from the time of introduction of BRAF and MEK inhibitors until death or date of censor (August 31, 2021). In the event of BRAF/MEK inhibitor–associated moderate or severe CTRCD, we collected data relating to discontinuation and dose alterations of BRAF and MEK inhibitors and the introduction of renin-angiotensin system inhibitors and beta-blockers.

### Echocardiography protocol and endpoints

Patients underwent transthoracic echocardiography before starting treatment with BRAF and MEK inhibitors, after 4 weeks, and at 3-month intervals thereafter. LVEF was calculated using the quantitative modified Simpson biplane method in accordance with the British Society of Echocardiography standards.^16^ GLS was analyzed retrospectively on transthoracic echo by 1 reader (C.G.) who was blinded to any prior echocardiographic measurements and patient visit number (GE EchoPAC software, version 204, revision 57.6). Endocardial borders were traced from 3 apical views (2-, 3-, and 4-chamber views). By tracing the endocardial border in end-systole, myocardial speckles were automatically tracked in subsequent frames and manually corrected if inadequate tracking was identified. GLS analysis was not performed if there was impaired regional tracking in >2 myocardial segments.

In accordance with the ICOS CTRCD definitions, BRAF/MEK inhibitor–associated mild asymptomatic CTRCD was defined as a relative worsening in GLS >15% compared with baseline with LVEF remaining ≥50%^6^; moderate asymptomatic CTRCD was defined as a new reduction in LVEF to 40% to 49% in association with either ≥10% absolute LVEF reduction from baseline or relative worsening in GLS by >15% compared with baseline.^6^ Severe asymptomatic CTRCD was defined as any new reduction in LVEF to <40%. Recovery was defined as an improvement of LVEF to at least 50%.

Interobserver variability assessment of GLS was carried out by K.M. (blinded to prior measurements and patient visit number) on 10% of the total number of scans analyzed. The interobserver intraclass coefficient was 0.92 (95% CI: 0.80-0.97).

### Statistical analysis

Baseline characteristics were summarized according to CTRCD status as defined by LVEF and GLS. All data are presented as mean ± SD or median (25th-75th percentiles [quartile (Q)1-Q3]) according to distribution. Categorical variables are presented as numbers and percentages. Histograms and Shapiro-Wilk tests were used to determine normality. The Student’s *t*-test, Mann-Whitney *U* test, or chi-square test was used to determine the association of baseline characteristics with the development of CTRCD. The chi-square test was used to assess the association between HFA/ICOS cardiotoxicity baseline risk category and the development of CTRCD. Repeated-measures mixed-effect models were used to examine the change in LVEF and the percentage change in GLS over time according to CTRCD status. Results are presented as the least squares mean with 95% CIs at each time point. Models were adjusted for baseline LVEF/GLS, visit, CTRCD status, and the interaction of CTRCD status and visit with a random intercept and slope per patient with an unstructured covariance structure. A normal distribution was used, and assumptions were tested by examining the plot of residuals and fitted values*.* The intraclass correlation coefficient was calculated using a 2-way mixed-effects model.

All analyses were performed using STATA version 17 software (StataCorp LLC). Statistical significance was defined as a 2-tailed *P* value <0.05 for all tests.

## Results

### Study population

**Figure 1:**
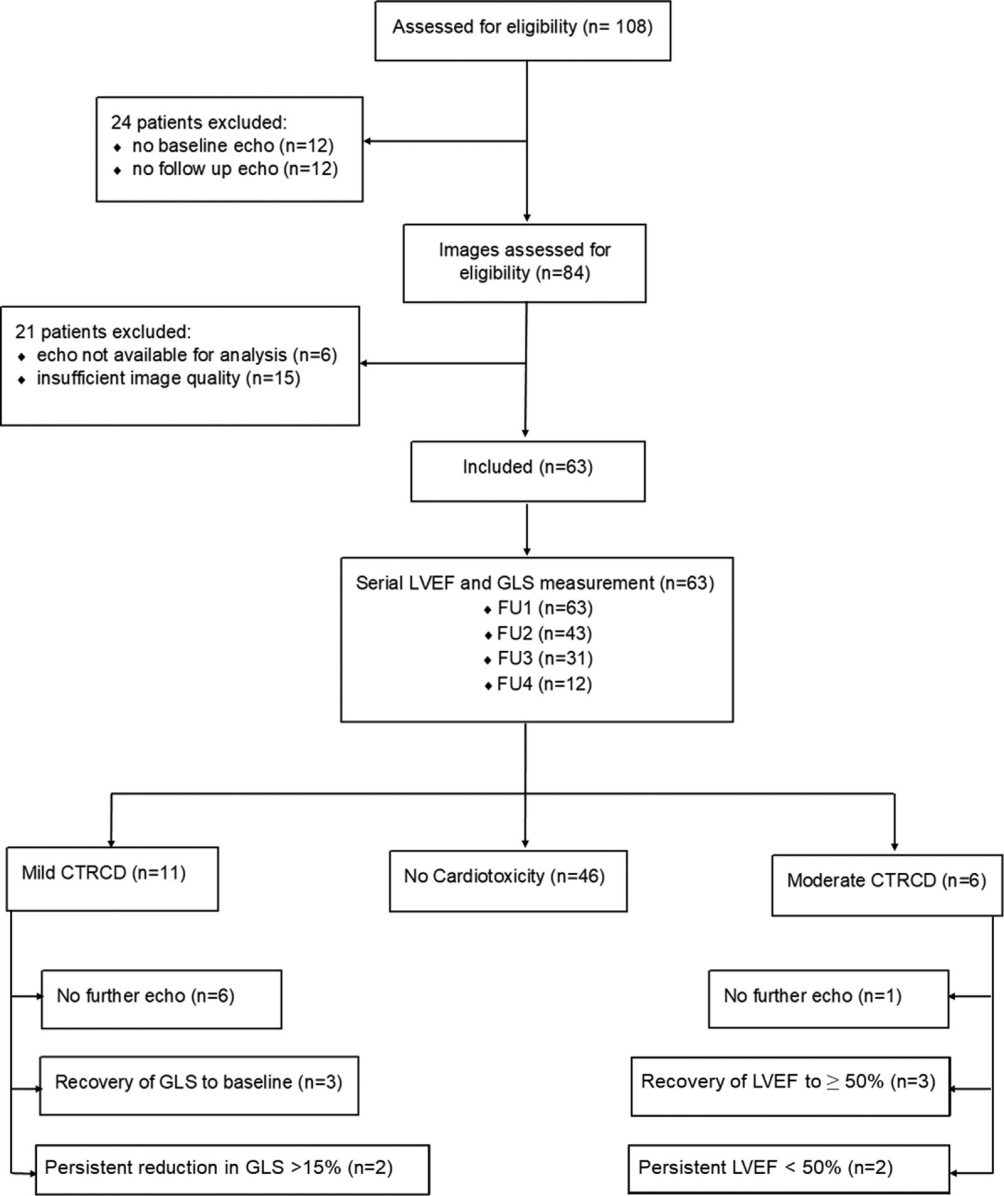
Patient Flowchart A flow diagram of participant progress throughout study follow-up. CTRCD = cancer therapy–related cardiac dysfunction; FU = follow-up; GLS = global longitudinal strain; LVEF = left ventricular ejection fraction.

A total of 63 patients were included as shown in the consort diagram (Figure 1). Fifty-four patients (86%) received a combination of dabrafenib and trametinib, and 9 patients (14%) received dabrafenib followed by encorafenib and binimetinib for disease progression. The median duration of treatment was 12 months (IQR [Q1-Q3]: 4-12 months). Twenty-eight patients (44%) were treated in the adjuvant setting, and 8 patients (13%) had previously received immunotherapy.

Baseline characteristics for the overall study population and according to the development of BRAF/MEK inhibitor–associated CTRCD are shown in Table 1. The mean age of the patients was 61 ± 14 years, and 62% were women. Three patients (5%) had a history of ischemic heart disease, 2 patients (3%) had a history of heart failure, and 22 patients (35%) had a history of hypertension. Baseline clinic systolic blood pressure (BP) was 138 ± 18 mm Hg, and diastolic BP was 79 ± 13 mm Hg. When stratified according to the HFA/ICOS cardiotoxicity risk tool, 46% of patients would be considered low risk, 40% medium risk, 11% high risk, and 3% very high risk (Table 2). The mean baseline LVEF and GLS were normal (LVEF: 63% ± 6%, GLS: −18.4% ± 2.5%). The baseline echocardiographic parameters are shown in Table 3.

**Table 1:** Baseline Characteristics by CTRCD Category

	All Patients (N = 63)	No CTRCD (n = 46)	Mild CTRCD (n = 11)	Moderate CTRCD (n = 6)	*P* Value
Age, y	61 ± 14	59 ± 15	65 ± 11	64 ± 14	0.49
Male	24 (38)	17 (37)	3 (27)	4 (67)	0.27
Indication					0.82
Adjuvant	28 (44)	21 (46)	4 (36)	3 (50)	
Metastatic	35 (56)	25 (54)	7 (64)	3 (50)	
BRAF/MEK type					0.095
Dabrafenib + trametinib	54 (86)	38 (83)	10 (91)	6 (100)	
Dabrafenib + trametinib + encorafenib + binimetinib	8 (13)	8 (17)	0 (0)	0 (0)	
Dabrafenib + encorafenib + binimetinib	1 (1)	0 (0)	1 (9)	0 (0)	
BMI >30 kg/m^2^	13 (21)	8 (17)	2 (18)	3 (50)	0.17
Smoker (current or previous)	26 (41)	17 (37)	6 (55)	3 (50)	0.51
Heart failure	2 (3)	1 (2)	0 (0)	1 (17)	0.13
IHD	3 (5)	2 (4)	0 (0)	1 (17)	0.29
HTN	22 (35)	16 (35)	3 (27)	3 (50)	0.64
Diabetes	10 (16)	8 (17)	1 (9)	1 (17)	0.79
eGFR <60 mL/min/1.73m^2^	6 (10)	4 (9)	1 (9)	1 (17)	0.82
Prior anthracycline	1 (2)	1 (2)	0 (0)	0 (0)	0.83
Prior immunotherapy	8 (13)	6 (13)	2 (18)	0 (0)	0.56
Prior BRAF/MEK inhibitors	1 (2)	1 (2)	0 (0)	0 (0)	0.83
Cardiovascular treatment					
Beta-blocker	5 (9)	2 (5)	0 (0)	3 (50)	<0.001
ACE inhibitor	9 (16)	7 (18)	0 (0)	2 (33)	0.22
Platelet inhibitor	4 (7)	2 (5)	2 (22)	0 (0)	0.15
Statin	15 (27)	10 (25)	2 (22)	3 (50)	0.41
Systolic BP, mm Hg	138 ± 18	139 ± 20	137 ± 12	138 ± 19	0.97
Diastolic BP, mm Hg	79 ± 13	80 ± 13	76 ± 15	80 ± 3	0.63

Values are mean ± SD or n (%).

*P* values represent the difference across groups.

ACE = angiotensin-converting enzyme; BMI = body mass index; BP = blood pressure; BRAF = rapidly accelerated fibrosarcoma B-type; CTRCD = cancer therapy–related cardiac dysfunction; eGFR = estimated glomerular filtration rate; HTN = hypertension; IHD = ischemic heart disease; MEK = mitogen-activated extracellular signal-regulated kinase.

**Table 2:** Patients Categorized According to HFA/ICOS Cardiotoxicity Baseline Risk Category

	All Patients	No CTRCD	Mild CTRCD	Moderate CTRCD
Low	29 (46)	22 (48)	5 (46)	2 (33)
Medium	25 (40)	18 (39)	5 (46)	2 (33)
High	7 (11)	5 (11)	1 (8)	1 (17)
Very high	2 (3)	1 (2)	0 (0)	1 (17)

Values are n (%).

CTRCD = cancer therapy–related cardiac dysfunction; HFA = Heart Failure Association; ICOS = International Cardio-Oncology Society.

**Table 3:** Baseline Echocardiographic Parameters by CTRCD Category

	All Patients (N = 63)	No CTRCD (n = 46)	Mild CTRCD (n = 11)	Moderate CTRCD (n = 6)	*P* Value
LVEF, %	63 ± 6	62 ± 6	66 ± 6	60 ± 4	0.074
GLS, %	−18.4 ± 2.5	−18.2 ± 2.0	−20.2 ± 2.8^a^	−17.2 ± 4.0	0.025
LVlDd, cm	4.3 ± 0.6	4.3 ± 0.6	4.1 ± 0.4	4.9 ± 0.6^a^	0.023
LVlDs, cm	2.9 ± 0.5	2.8 ± 0.5	2.6 ± 0.2	3.3 ± 0.4^a^	0.028
IVSd, cm	1.0 ± 0.2	1.0 ± 0.2	1.0 ± 0.2	1.0 ± 0.2	0.90
LA area, cm^2^	16.8 ± 4.0	16.7 ± 3.5	16.0 ± 3.2	19.8 ± 7.8	0.18
E/A ratio	1.0 ± 0.3	1.0 ± 0.3	1.0 ± 0.3	1.1 ± 0.5	0.63
TR Vmax, m/s	2.3 ± 0.4	2.4 ± 0.4	2.2 ± 0.1	2.0 ± 0.1	0.19
RV s’, cm/s	12.4 ± 2.1	12.4 ± 2.2	13.4 ± 1.8	11.2 ± 1.7	0.16
TAPSE, cm	2.2 ± 0.4	2.2 ± 0.4	2.3 ± 0.5	2.0 ± 0.3	0.47
E/e’ ratio	7.4 ± 2.1	7.3 ± 2.0	7.1 ± 2.2	8.8 ± 2.7	0.31

Values are mean ± SD.

*P* values represent the difference across groups.

GLS = global longitudinal strain; IVSd = interventricular septum diameter end-diastole; LA = left atrium; LVEF = left ventricular ejection fraction; LVlDd = left ventricular diameter (diastole); LVlDs = left ventricular diameter (systole); RV s’ = tissue Doppler peak right ventricular systolic velocity; TAPSE = tricuspid annular plane systolic excursion; TR Vmax = maximal tricuspid regurgitation velocity.

a *P <* 0.05 for pairwise comparison vs no CTRCD group.

### BRAF and MEK inhibitor–associated CTRCD

#### Any cardiotoxicity

Seventeen patients (27%) developed CTRCD during BRAF/MEK inhibitor treatment. No patient developed severe asymptomatic CTRCD (LVEF <40%) or symptomatic heart failure. There were no significant differences in baseline characteristics between those who developed CTRCD and those who did not.

#### Mild CTRCD

**Figure 2:**
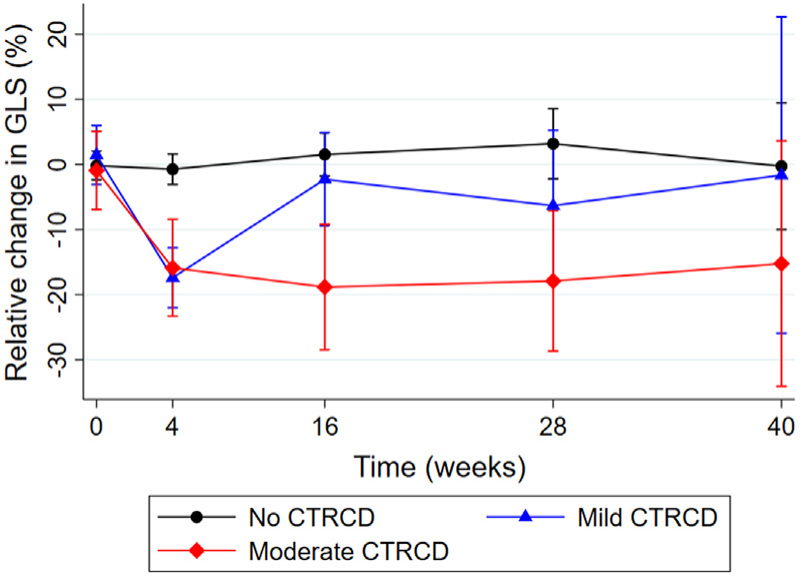
GLS During Treatment With BRAF and MEK Inhibitors The black line represents no CTRCD, the blue line represents mild CTRCD, and the red line represents moderate CTRCD. Circles, triangles, and diamonds indicate the least squares mean, and whiskers indicate 95% CIs. BRAF = rapidly accelerated fibrosarcoma B-type; MEK = mitogen activated extracellular signal-regulated kinase; other abbreviations as in Figure 1.

Eleven patients (17%) developed mild CTRCD, which was defined as a worsening in GLS >15% relative to baseline with LVEF remaining ≥50%. There was a nonsignificant tendency for higher baseline LVEF in patients who developed mild CTRCD in comparison to those who did not develop cardiotoxicity (LVEF: 66% ± 6% vs 62% ± 6%; *P =* 0.051), and baseline GLS was significantly improved in those who developed mild CTRCD compared with those with no CTRCD (GLS: −20.2% ± 2.8% vs −18.2% ± 2.0%; *P =* 0.008). In patients who developed mild CTRCD, the median relative decrease in GLS was 21.5% (Q1-Q3: 17.8%-22.6%) with a median time to onset of 2 months (Q1-Q3: 2-4 months). The first incidence of mild CTRCD occurred at 4 weeks in 8 patients (73%), at 4 months in 2 patients (18%), and at 7 months in 1 patient (9%). The change in GLS during follow-up is shown in Figure 2. Of the 11 patients with mild CTRCD, 6 patients had no further echocardiography because treatment was stopped for noncardiovascular reasons following the visit with a decline in GLS, there was recovery to baseline in 3 patients, and there was a persisting >15% reduction in GLS in 2 patients. Of those with mild CTRCD who had subsequent echocardiography, none progressed to moderate or severe CTRCD. No patients developed symptomatic heart failure.

#### Moderate CTRCD

**Figure 3:**
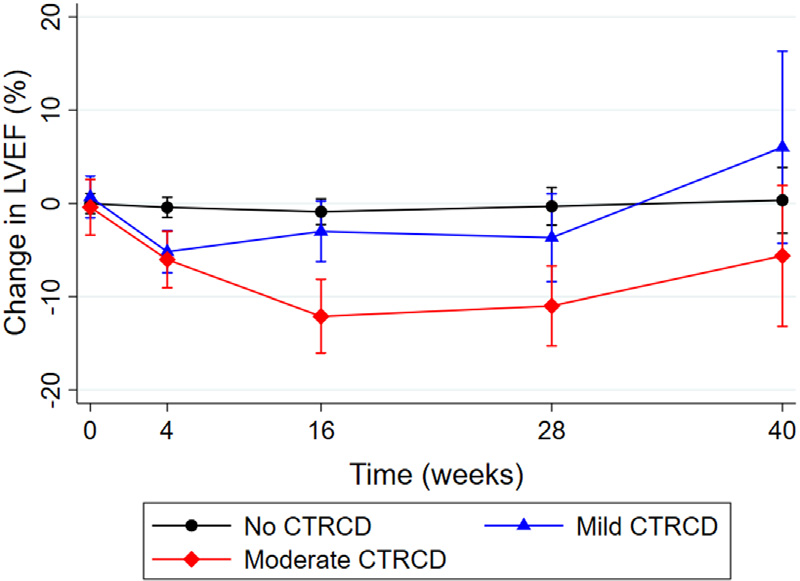
Left Ventricular Ejection Fraction During Treatment With BRAF and MEK Inhibitors The black line represents no CTRCD, the blue line represents mild CTRCD, and the red line represents moderate CTRCD. Circles, triangles, and diamonds indicate the least squares mean, and whiskers indicate 95% CIs. Abbreviations as in Figures 1 and 2.

Six patients (10%) developed moderate CTRCD as defined by a reduction in LVEF ≥10% from baseline to an absolute value 40%-49%. No patients were classified as moderate CTRCD on the basis of a smaller change in LVEF with a concomitant decrease in GLS. There was no significant difference in baseline LVEF or GLS between patients who developed moderate CTRCD and those with no CTRCD (LVEF: 60% ± 4% vs 62 ± 6%; *P =* 0.41; GLS: −17.2% ± 4.0% vs −18.2% ± 2.0%; *P =* 0.36). However, patients who developed moderate CTRCD had significantly larger left ventricular systolic and diastolic diameters at baseline than those who did not develop CTRCD (Table 3). The median decrease in LVEF in patients with moderate CTRCD was 15% (Q1-Q3: 12%-16%) with a median time to onset of 4 months (Q1-Q3: 1-6 months). The first incidence of moderate CTRCD occurred at 4 weeks in 3 patients (50%), at 4 months in 2 patients (33%), and at 7 months in 1 patient (17%). The changes in LVEF during follow-up are shown in Figure 3. All patients with moderate CTRCD also had a relative reduction in GLS of >15%, but this change in GLS did not precede the diagnosis of moderate CTRCD in any patient.

#### HFA/ICOS baseline risk category and subsequent CTRCD

Of the patients who developed mild CTRCD, the majority met the criteria for low or medium risk of cardiotoxicity according to the HFA/ICOS score (5 patients [46%] low risk and 5 patients [46%] moderate risk). One patient met the baseline criteria for high risk, and there were no very high-risk patients. Of those who developed moderate CTRCD, 2 patients (33%) were in the HFA/ICOS low-risk group, 2 patients (33%) were in the medium-risk group, 1 patient (17%) was in the high-risk group, and 1 patient (17%) was in the very high–risk group (Table 2). In patients who developed any severity of CTRCD (mild or moderate), 24% were in the HFA/ICOS low-risk category, 28% in the medium-risk category, 29% in the high-risk category, and 50% were considered to be very high risk, but only 2 patients were in the very high–risk category. These differences were not statistically significant (*P =* 0.603), likely because of the small sample size.

#### Management and clinical course of CTRCD

Because the diagnosis of mild CTRCD was made retrospectively for the purposes of this analysis, no patient with mild CTRCD had a change in cancer treatment or cardiovascular therapies on the basis of a decline in GLS. However, in patients with mild CTRCD, it was noted that 3 patients had a BRAF inhibitor and MEK inhibitor dose reduction by 1 dose level, and 2 patients stopped treatment because of noncardiovascular toxicities. In patients with moderate CTRCD, the MEK inhibitor was temporarily interrupted in 4 cases, and both BRAF and MEK inhibitors were interrupted in the other 2 cases because of concurrent noncardiovascular toxicities. Four patients were referred to a cardiologist for consultation. One patient was commenced on an angiotensin-converting enzyme inhibitor, and another patient already receiving angiotensin-converting enzyme inhibitor therapy had the dose of this increased. No patients were started on beta-blockers.

Of the 6 patients with moderate CTRCD, LVEF recovered to an absolute value above 50% in 3 patients with times to recovery of 1, 8, and 12 months. LVEF did not recover in 2 patients, and the remaining patient did not have further echocardiography because treatment was stopped after the visit at which CTRCD was identified. No patients progressed to severe asymptomatic CTRCD or symptomatic heart failure. BRAF/MEK inhibitor treatment was successfully restarted at a reduced dose in 4 patients and permanently discontinued in 2 patients (in 1 case because the intended 12-month BRAF/MEK inhibitor treatment period had elapsed, and, in the other case, BRAF/MEK inhibitor was not restarted because of other noncardiovascular toxicities). No patient had recurrence of BRAF/MEK inhibitor–associated CTRCD after treatment reintroduction.

### CTRCD and other cardiovascular adverse events

Other cardiovascular adverse events did not occur more frequently in patients who developed CTRCD than those who did not. During treatment, 5 patients had a venous thromboembolic event (3 of these had no CTRCD, and 2 had mild CTRCD), 2 patients had arrhythmias (1 atrial flutter, and 1 conduction disorder requiring a pacemaker; none of these had CTRCD), and 2 patients had unexplained syncope. Both patients had normal resting and multiday electrocardiograms (1 of these had no CTRCD, and 1 had mild CTRCD).

## Discussion

To the best of our knowledge, this is the first study to report longitudinal assessment of left ventricular systolic function using both LVEF and GLS after treatment with BRAF and MEK inhibitors. It is also the first to incorporate the HFA/ICOS baseline cardiotoxicity risk stratification tool^15^ as recommended by the 2022 ESC Cardio-Oncology guidelines.^14^ The principal finding of this study is that 27% of patients in this cohort developed BRAF and MEK inhibitor–associated CTRCD with 17% meeting the criteria for mild CTRCD and 10% meeting the definition of moderate CTRCD. The majority of patients who developed CTRCD would have been considered as low or medium risk for the development of cardiotoxicity using the HFA/ICOS baseline risk stratification tool.

The 10% incidence of BRAF/MEK inhibitor–associated moderate CTRCD is in keeping with the incidence of LVSD of 2% to 12% reported from clinical trials in which LVSD was defined as a reduction in LVEF ≥10% from baseline to an absolute value <50%.^17^, ^18^, ^19^, ^20^, ^21^, ^22^ This is slightly lower than the incidence of 13.6% in another retrospective cohort; however, that study defined LVSD as a reduction in LVEF ≥10% from baseline to a value <55%.^23^ Although a reduction in LVEF was fairly common in our cohort, none of these patients developed symptomatic heart failure. Patients who developed moderate CTRCD had larger baseline left ventricular dimensions than those who did not develop CTRCD, but no other baseline characteristic was associated with subsequent moderate CTRCD in our cohort.

In patients who had a reduction in LVEF, there was a heterogenous approach to management with only 2 patients receiving angiotensin-converting enzyme inhibitor therapy and no patients receiving beta-blockers. This may reflect the lack of contemporary guidelines for the management of BRAF and MEK inhibitor–associated cardiotoxicity. Despite this low rate of cardioprotective medication initiation, the majority of patients were successfully re-established on BRAF/MEK inhibitor therapies with no recurrence of left ventricular systolic impairment. Given the relatively small sample size, it is not possible to draw firm conclusions about the reversibility of BRAF/MEK inhibitor–associated CTRCD. However, our results suggest a reversible component in keeping with reports from previous observational studies^23^ and similar to CTRCD associated with other non–BRAF/MEK inhibitor tyrosine kinase inhibitor anticancer therapies.^24^

GLS assessment is a marker of myocardial dysfunction and can be used to detect early subclinical ventricular dysfunction.^7^ Most publications relating to its use in this context refer to monitoring recommendations and practice in patients with breast cancer receiving anthracycline or trastuzumab therapy. The endorsement of recent GLS-based definitions of CTRCD in the recent ESC cardio-oncology guidelines as well as the recommendation for GLS to be measured in all patients with cancer having an echocardiogram^14^ may lead to its wider use. However, the long-term implications of impaired GLS remain much less well established than they are for reductions in LVEF. This is especially true for patients without breast cancer and those treated without anthracycline or trastuzumab. Unsurprisingly, the incidence of mild, GLS-defined cardiotoxicity was greater than the incidence of moderate, LVEF-defined cardiotoxicity in our cohort at 27% and 10%, respectively. The median time to a reduction in GLS was shorter than the time to a reduction in LVEF as may be expected, but new impairment in GLS (mild CTRCD) was not associated with a subsequent decline in LVEF (moderate CTRCD). Patients who met the criteria for mild CTRCD had better baseline GLS than those who did not develop CTRCD, and this raises the possibility of regression to the mean over time rather than genuine new impairment of GLS or “true” cardiotoxicity. For these reasons, the clinical relevance of mild cardiotoxicity criteria remains to be established in this patient group, and there is a potential risk of overdiagnosis of CTRCD. Indeed, inappropriate interruption of important anticancer therapy in the setting of mild CTRCD would be inadvisable, although closer monitoring may be reasonable. Larger prospective studies are needed to determine any association between relative and absolute changes in GLS and cardiovascular outcomes for patients treated with BRAF and MEK inhibitors.

In this cohort, CTRCD occurred at any point during the course of treatment with BRAF and MEK inhibitors (Central Illustration). Of those who developed any CTRCD, the majority (82%) would have been considered as low or medium risk at baseline for the subsequent development of cardiotoxicity using the HFA/ICOS risk assessment tool. For those who developed moderate CTRCD, 66% would have been considered at low or medium risk of cardiotoxicity. Therefore, current recommendations in the ESC cardio-oncology guidelines to consider echocardiography every 4 months in HFA/ICOS-determined high-risk patients only^14^ may miss CTRCD in low- and medium-risk patients. Further prospective studies are required to determine the echocardiographic surveillance strategies required for these therapies.

### Study limitations

Troponin and N-terminal pro–B-type natriuretic peptide were not routinely measured in this cohort and therefore could not be used to calculate patients’ risk score. These markers are recommended in the assessment of cardiovascular toxicity from cancer therapies.^25^ Similar to GLS, these biomarkers have not been rigorously examined to inform treatment and surveillance strategies specifically in patients treated with BRAF/MEK inhibitors. Unfortunately, longitudinal BP measurements were not available in every patient’s clinical record and therefore were not included in our evaluations. Our study has other limitations, including a relatively small sample size giving rise to limited statistical power. We did not correct for multiple pairwise comparisons of baseline characteristics; therefore, these statistically significant results are preliminary and require validation in larger cohort studies. There was also a high percentage of drop out with only 19% of patients assessed at the final time point (week 40). The main reasons for dropout were discontinuation of treatment or death. Therefore, our study cannot draw conclusions on delayed BRAF and MEK inhibitor–associated CTRCD. However, our data reflect a real-world population and fill an important gap in an area with a very limited evidence base.

## Conclusions

BRAF and MEK inhibitor–associated CTRCD is common. CTRCD can manifest at any time during treatment with BRAF and MEK inhibitors and, in this cohort, occurred most frequently in patients who would be considered low and medium risk for the development of cardiotoxicity. This suggests that prospective echocardiographic follow-up should be considered in patients treated with BRAF/MEK inhibitors until better risk stratification tools are developed and validated. Although mild CTRCD was common, prospective studies are required to determine the clinical relevance of changes in GLS in patients treated with BRAF and MEK inhibitors.

Perspectives **COMPETENCY IN MEDICAL KNOWLEDGE:** In patients with melanoma treated with BRAF and MEK inhibitors, the development of CTRCD is common. Patients with larger left ventricular dimensions before starting BRAF and MEK inhibitors were at increased risk for CTRCD, but no other characteristics were closely associated with the risk for CTRCD. CTRCD occurred most frequently in patients considered to be at low or medium baseline risk for the development of cardiotoxicity, suggesting the utility of the HFA/ICOS risk stratification tool appears to be limited in this group. Our results suggest that prospective echocardiography follow-up should be considered in patients treated with BRAF and MEK inhibitors until better risk stratification tools are developed and validated.

**TRANSLATIONAL OUTLOOK:** Further research is needed to develop better risk stratification tools and to determine the long-term consequences of CTRCD (particularly mild CTRCD) in patients with melanoma treated with BRAF and MEK inhibitors.

## Funding Support and Author Disclosures

Drs Glen and Lang are supported by an unrestricted grant from Roche Diagnostics. Drs Petrie and Lang are supported by a British Heart Foundation Centre of Research Excellence grant (RE/18/6/34217). Dr Petrie has received research funding from Boehringer Ingelheim, Roche, SQ Innovations, AstraZeneca, Novartis, Novo Nordisk, Medtronic, Boston Scientific, Pharmacosmos, and 3R LifeSciences; and is a consultant and on clinical trials committees for Boehringer Ingelheim, Novartis, Roche, Corvia, AstraZeneca, Novo Nordisk, Medtronic, Abbvie, Bayer, Takeda, Cardiorentis, Pharmacosmos, and Siemens. Dr Lang has received speaker fees from Roche, Pfizer, and Novartis.
