# Supplementary material for: Cardiotoxicity of BRAF/MEK Inhibitors: A Longitudinal Study Incorporating Contemporary Definitions and Risk Scores
Source: JACC CardioOncol. 2023 Jun 6;5(5):628–37. doi: 10.1016/j.jaccao.2023.04.004 (PMC10635885; doi:10.1016/j.jaccao.2023.04.004)
Supplement: Supplemental Table 1 [file mmc1.docx]

# **Cardiotoxicity of BRAF/MEK Inhibitors:**

# **A Longitudinal Study Incorporating Contemporary Definitions and Risk Scores**

**Supplemental Table 1**

HFA-ICOS Baseline cardiovascular risk stratification proforma for combination RAF and MEK inhibitors ^15^

|  | |
| --- | --- |
| Risk Factor | **Score** |
| *Previous cardiovascular disease* |  |
| Heart failure or cardiomyopathy | Very high |
| Myocardial infarction or CABG | High |
| Stable angina | High |
| Severe valvular heart disease | High |
| Borderline LVEF 50-54% | Medium^2^ |
| Arrhythmia^a^ | Medium^1^ |
| *Cardiac biomarkers (when available)* |  |
| Elevated baseline troponin^b^ | Medium^2^ |
| Elevated baseline BNP or NT-proBNP^b^ | Medium^2^ |
| *Demographic and cardiovascular risk factors* |  |
| Age ≥ 65 years | Medium^1^ |
| Hypertension^c^ | Medium^2^ |
| Diabetes Mellitus^d^ | Medium^1^ |
| Chronic kidney disease^e^ | Medium^1^ |
| *Previous cardiotoxic cancer treatment* |  |
| Prior anthracycline exposure^f^ | High |
| Prior radiotherapy to left chest or mediastinum | Medium^2^ |
| *Lifestyle risk factors* |  |
| Current smoker or significant smoking history | Medium^1^ |
| Obesity (BMI > 30kg/m^2^) | Medium^1^ |
| BMI, body mass index; BNP, brain natriuretic peptide; CABG, coronary artery bypass graft; LVEF, left ventricular ejection fraction; NT-proBNP, N-terminal pro-brain natriuretic peptide  Low risk = no risk factor or one medium^1^ risk factor  Medium risk = medium risk factors with a total of 2-4 points  High risk = medium risk factors with a total of ≥ 5 points OR any high risk factor  Very high risk = any very high risk factor  ^a^ Atrial fibrillation, atrial flutter, ventricular tachycardia, or ventricular fibrillation.  ^b^ Elevated above the upper limit of normal for local laboratory reference range.  ^c^ Systolic blood pressure >140mmHg or diastolic blood pressure >90 mmHg, or on treatment.  ^d^ Glycated hemoglobin >7.0% or >53 mmol/mol, or on treatment.  ^e^ Estimated glomerular filtration rate <60 mL/min/1.73m^2^.  ^f^ Previous malignancy | |
